# Comparing the accuracy of Pipelle versus hysteroscopy and curettage in the diagnosis of chronic endometritis in women with recurrent implantation failure: A prospective cross-sectional study

**DOI:** 10.1371/journal.pone.0319294

**Published:** 2025-03-21

**Authors:** Elaheh Pahlevan Falahy, Mohammad-Taha Pahlevan-Fallahy, Fatemeh Keikha

**Affiliations:** 1 Department of Obstetrics and Gynecology, Tehran University of Medical Sciences, Tehran, Iran; 2 Health Reproductive Research Center, Imam Khomeini Complex Hospital, Tehran University of Medical Sciences, Tehran, Iran; 3 School of Medicine, Tehran University of Medical Sciences, Tehran, Iran; King Saud University / Zagazig University, EGYPT

## Abstract

**Objectives:**

Chronic endometritis (CE) is defined as chronic inflammation in the endometrium; when treated, implantations significantly improve. The standard test for CE confirmation is an endometrial biopsy, but the appropriate sampling method needs to be clarified. We conducted this study to compare pipelle biopsy and hysteroscopy with curettage.

**Study design:**

This is a prospective cross-sectional study with all (40 patients) RIF patients under 40 referred to our tertiary center between December 2021, and December 2022 who underwent pipelle biopsy and hysteroscopy with curettage between days twelve to fifteen of their menstruation cycle. We then compared the diagnostic accuracy, demographics, and previous IVF history between the CE and non-CE groups.

**Results:**

Patients had a mean age of 34 ( ± 5.4) years and BMI of 25.8 ( ± 3.6). Thirteen patients (32.5%) were diagnosed with CE. There was no significant difference between CE and non-CE groups regarding maternal or paternal age, BMI, number of IVFs and embryos, and interval from the last IVF. Pipelle biopsy had 100% accuracy for CE diagnosis, while hysteroscopy with curettage had a sensitivity of 92.3% (95% CI: 77.8% - 100%) and specificity of 100%. Based on McNemar’s test, the two sampling methods had no significant difference (P = 1.0 and 0.317, respectively).

**Conclusion:**

There is no significant difference between the two methods in the diagnosis accuracy of CE in RIF patients. Since pipelle is more cost-effective and has fewer complications than hysteroscopy with curettage, pipelle biopsy may replace curettage for CE diagnosis.

## Introduction

Assistive reproductive techniques (ART) have had impressive advancements since the first in vitro fertilization (IVF) in 1978. Nowadays, a wide variety of ART options are available to couples struggling with infertility, including IVF, intracytoplasmic sperm injection (ICSI), gamete intrafallopian transfer (GIFT), and zygote intrafallopian transfer (ZIFT). These progresses have led to higher success rates and lower risks for patients undergoing ART procedures. There are various influential factors regarding the success rate of IVF, including the quality and number of embryos transferred, the age and health of the patient, and the expertise of the IVF clinic staff. Despite the use of advanced technologies such as preimplantation genetic testing (PGT), implantation remains the bottleneck for IVF procedures, limiting the success rate of IVF procedures. This can be very frustrating for patients, who have invested their time, money, and hopes into ART procedures.

Implantation is a complex process during which the blastocyte gets embedded in the endometrial stroma. It is well-known that implantation success depends on embryo quality, uterine integrity, and receptivity of the endometrial cavity [1]. Embryo quality is the most crucial component affecting implantation success, which can be controlled using PGT; uterine abnormalities such as polyps, myomas, and fibroids can also be corrected to increase fertility success rates. In previous studies, RIF is defined as failure to conceive after three cycles of IVF with good-quality fresh or frozen embryos in women < 35 and four cycles in women > 35 [2,3]. Lack of endometrial receptivity is one of the most important reasons for implantation failure. Endometrial receptivity is “the complex process that allows the embryo to attach, invade, and develop” [4]. Its window extends three to six days within the secretory phase in most normal women, and anatomical and inflammatory conditions can narrow the fertility window or lead to infertility [4].

Chronic endometritis (CE) is the persistent inflammation or infection of the endometrial lining. It can be challenging to diagnose since most patients with CE are asymptomatic or present with mild symptoms. Most cases of CE are suspected when RIF, recurrent pregnancy loss (RPL), chronic pelvic pain (CPP), dyspareunia, abnormal uterine bleeding, or persistent vaginal discharge persists, or any abnormalities are seen in the uterine lining [3,5]; the prevalence of CE in RIF patients have been estimated between 14 to 67.5 percent [3,6–12]. The exact impact of CE on reproductive function is controversial. Still, since the levels of proinflammatory cytokines such as IL-6, IL-1β, and TNF- α and the secretion of IgM, IgG, and IgA antibodies are increased in women with CE, it may negatively affect the endometrium, making it less suitable for implantation [3,13–15].

CE diagnosis is confirmed histologically by finding plasma cell infiltrates in endometrial biopsies [4,6,16]. CE treatment typically consists of antibiotics and anti-inflammatory drugs to treat the infections, reduce the inflammations, and promote the healing of the endometrium. CE can be seen visualized in hysteroscopy as mucosal edema, endometrial hyperemia, and micro-polyps [3,17,18]. The best test for confirmation of CE diagnosis is endometrial biopsy [6,9,17,19–22]. Various methods for endometrial biopsy exist, such as pipelle, dilation & curettage (D&C), and hysteroscopy. Compared to pipelle, hysteroscopy, and D&C are relatively invasive techniques requiring the patients to undergo local or general anesthesia and might be considered even harmful in patients suffering from infertility. They have longer recovery times than pipelle biopsy, are more painful, and are associated with a higher risk of complications such as uterine perforation, bleeding, and infection [23,24]. The diagnostic performance of pipelle and hysteroscopy have been compared in few studies for detection of CE in primary RIF patients. In this study, we tried to compare the diagnostic accuracy of pipelle biopsy with hysteroscopy with curettage for the detection of CE in RIF patients and measure the prevalence of CE in RIF patients, and also estimate the prevalence of CE in RIF patients.

## Materials and methods

### Study design and settings

The study was designed in accordance with the recommendations of STARD-2015 guideline for reporting diagnostic accuracy studies [25]. All patients with primary infertility diagnosis and RIF who were referred to our center between from December 2021 and December 2022 were included in our prospective cross-sectional study. Those who were pregnant or were later determined to have become pregnant, had undergone previous uterine surgery or had any uterine abnormality, or could not tolerate or had contraindications to pipelle biopsy or hysteroscopy and curettage, or did not consent to be part of the study were excluded from this study. The patients who suffered from secondary infertility were excluded due to different pathophysiology of disease. Also, the patients had consumed antibiotics in the month before the study were excluded since antibiotics use are known to alter the endometrial biome and inflammatory responses [26]. All patients undergoing the two sampling methods were tested for antiphospholipid antibodies, were negative, and had normal karyotypes. All participants underwent pipelle biopsy and hysteroscopy with curettage. Data, including demographic information, history of previous IVFs, clinical history, and pathology results, were collected from the medical records.

### Ethics

Patients were informed of the study procedure, and written informed consent was acquired from all included patients to participate in the study and publish the results. The study protocol was reviewed and ethically approved by the research ethics committee of Imam Khomeini Hospital Complex, Tehran University of Medical Sciences, Tehran, Iran (IR.TUMS.IKHC.REC.1400.357).

### Sample size

Previous studies have estimated the prevalence of CE in RIF patients between 2.8 and 67.5% [3,6–11]. The exact sensitivity and specificity of pipelle in diagnosing CE is still unknown. However, some studies have compared pipelle with hysteroscopy in diagnosing endometrial cancer and have reported sensitivities and specificities between 80 and 98 percent [20,21,27,28]. Sample size calculation was done considering the desired power (1- β) of 0.8 and significance level (α) of 0.05 using the formula below considering the variability in the estimated prevalence of CE in RIF patients in different studies. The minimum sample size required was calculated to be 37. Since there was no data on the diagnostic accuracy of pipelle, we considered conducting a pilot-study sample size of 40 for that. Finally, we chose a minimum sample size of 40 to satisfy both study objectives.


$$n=\frac{z_{1-\frac{\alpha }{2}}^{2}pq}{d^{2}}$$

### Intervention

Patients who were eligible to be included, came to the hospital on days 12-15 of their menstrual cycles. The patients underwent a blind biopsy using a pipelle device (Medbar, Turkey). Then, they were placed on a gynecological bed in a lithotomy position and received general anesthesia two days later and underwent rigid hysteroscopy (KARL STORZ, Germany) using a 30° lens and normal saline media; a thin, flexible tube with a camera and a light source is inserted into the uterus through the cervix to inspect the uterine cavity first; the anterior and posterior walls of the uterus were observed. Then, the uterine lining was visualized and inspected for any signs of CE (mucosal edema, endometrial hyperemia, and micro-polyps). The isthmus, uterine walls, fundus, external cervical os, and tubal ostia were inspected for any irregularities. The signs when visually inspecting the uterus were the existence of micro polyps (pedunculated and vascularized, < 1 mm); and the presence of “strawberry aspect”, which is an area of hyperemia in the endometrium with a white central point [3,29]. The suspected areas were then sampled using a sharp curette. The acquired samples were preserved in neutral formalin and sent to the pathology lab. Patients reported positive for CE according to the criteria mentioned later in the definitions section, were then treated with a 14-day course of oral doxycycline (100 mg twice daily). The visual findings for inspection of CE and the timing of visual inspection and interval between sampling in our study was replicated from previous credible studies [3,30–32]. We chose to sample the endometrial cavity seven days after the end of menstruation, since in this early follicular phase the endometrium can be visualized without the interference of endometrial thickened tissue [31].

### Definitions

Primary infertility is the inability to achieve a clinical pregnancy after at least twelve months of coitus without any underlying pathology. According to the study by Bouet et al., RIF was defined as having at least three cycles of IVF-ET with good-quality fresh or frozen embryos without achieving any clinical pregnancy in women < 35 and four cycles of IVF with at least four good quality fresh or frozen embryos in women > 35 [3]. Different studies considered 1-5 plasma cells per high-power field (HPF) for CE confirmation [33]. In our study, the biopsy samples were considered positive for CE if **five or more** plasma cells were seen per HPF to reduce false positives. If the patient had five or more plasma cells per HPF **in at least one of two samples collected**, the patient was diagnosed with CE. Patients who were not diagnosed with CE were than categorized into the secretory and proliferative endometrium and disorder proliferative based on the pathology report and clinical history and examinations [34]. All biopsies were evaluated by an expert pathologist specialized in endometrial pathology in our center. An embryo was considered good quality if it reached the blastocyte stage or grew to at least six cells on day three with a minimum grading of 3.

### Study objectives

Our primary objective was to compare the diagnostic accuracy of pipelle and hysteroscopy with curettage in diagnosing CE. The secondary objective of our study was to estimate the prevalence of CE in RIF patients.

### Statistical analysis

For continuous variables, data were reported as mean ±  SD; sensitivity, specificity, positive and negative predictive value (PPV and NPV, respectively), and accuracy for pipelle and hysteroscopy were calculated according to the criteria for CE diagnosis. Kolmogorov-Smirnov (K-S) test was used to assess the normality of data for continuous variables. If the variable had normal distribution, independent samples T-test was used to compare the CE and non-CE groups and ANOVA was used to compare the four groups. When the variable did not have a normal distribution, Mann-Whitney U test was used instead of independent samples T-test and Kruskal-Wallis H test was used instead of ANOVA. Categorical variables were analyzed using chi-square test. Stuart-Maxwell’s test (marginal homogeneity) was used to compare pipelle and hysteroscopy with curettage diagnostic accuracy for the four possible pathology results; proliferative endometrium, secretive endometrium, proliferative disorder, and chronic endometritis. McNemar’s test was used to test the diagnostic accuracy of both methods with the ultimate diagnosis, grouped into CE patients and non-CE patients. Statistical analysis was done using SPSS 26, and P <  0.05 was considered statistically significant.

## Results

Forty women were included in this study. Their age ranged from 21 to 45 years (mean ±  SD: 34.0 ±  5.4). Their spouses’ age ranged from 21 to 54 (mean ±  SD: 31.9 ±  6.6). The patient’s BMI was in the range of 16.36-32.81 (mean ±  SD: 25.8 ±  3.6). Duration of Infertility in patients was in ranged from 2 to 20 years (mean ±  SD: 6.3 ±  4.1). In each cycle, one or two embryos were transferred. Patients underwent 3 - 5 (mean ±  SD: 3.18 ±  0.44) rounds of IVF. The number of embryos transferred for each patient ranged from 4-11 (mean ±  SD: 6.0 ±  1.6). The interval from their last IVF was 8.9 ±  5.5 months. All embryos had good quality (100%). All patients had primary infertility, and none of the patients or samples were lost during the research. None of the samples acquired were marked as inadequate. 37 (92.5%) of patients said they would prefer only a pipelle biopsy, and three (7.5%) preferred hysteroscopies with curettage if they were to choose one method.

According to the diagnostic criteria mentioned before, 13 (32.5%) of patients’ samples reported secretory endometrium; 13 (32.5%) were proliferative endometrium, one (2.5%) reported proliferative disorder and 13 (32.5%) had chronic endometritis.

When compared, there was no significant difference between any of the four groups in patient age, spouse age, duration of infertility, BMI, the interval from the last IVF, the total number of embryos, and IVF (P > 0.05) (Table 1). Since the distribution was not normal in the duration of infertility, the number of embryos and IVFs, and the interval from the last IVF, they were analyzed using non-parametric tests, as stated in the methods section. A descriptive summary of the patients categorized based on their final diagnoses can be seen in Table 2.

**Table 1:** Comparison of variables between CE and non-CE patients. Data are reported as mean (± SD).

Variable	CE (n = 12)	Non-CE (n = 28)	P-Value
Maternal age	34.5 ( ± 4.8)	33.9 ( ± 5.7)	0.751
Paternal age	40.5 ( ± 5.9)	38.5 ( ± 6.9)	0.386
BMI	26.7 ( ± 4.1)	25.5 ( ± 3.4)	0.309
Duration of infertility (years)	6.3 ( ± 4.0)	6.3 ( ± 4.3)	0.493
Total embryos transferred	5.5 ( ± 1.5)	6.2 ( ± 1.6)	0.252
Total IVF cycles	3.1 ( ± 0.4)	3.1 ( ± 0.5)	1.00
Interval since last IVF (months)	7.7 ( ± 3.8)	9.4 ( ± 6.2)	0.919

**Table 2:** A descriptive summary of patients categorized based on the final pathology report. Data were reported as mean ±  SD and no SD was reported in the third column since there was only one patient in that group.

Variables	Chronic endometritis (n = 13)	Disorder Proliferative (n = 1)	Proliferative endometrium (n = 13)	Secretory endometrium (n = 13)	Total (n = 40)
Patient Age	34.76 ± 4.7	21.0	34.46 ± 5.4	34 ± 5.4	34.07 ± 5.4
Spouse Age	40.69 ± 5.7	21.0	39.38 ± 6.8	38.61 ± 5.5	39.1 ± 6.5
Duration of Infertility (years)	7.19 ± 5	5.0	4.8 ± 2.3	7.07 ± 4.6	6.32 ± 4.1
Number of Embryos Transferred from IVF	5.53 ± 1.4	4.0	5.84 ± 0.8	6.76 ± 2	6 ± 1.6
Number of IVF	3.15 ± 0.3	3.0	3.15 ± 0.3	3.23 ± 0.5	3.17 ± 0.4
Last IVF Month	8.23 ± 4	5.0	8.38 ± 5	10.46 ± 7.3	8.92 ± 5.5
BMI	26.81 ± 3.9	25.3	25.85 ± 4.5	24.9 ± 1.9	25.84 ± 3.6

Based on the diagnostic criteria, pipelle biopsy had 100% sensitivity and 100% specificity for CE diagnosis. Pipelle biopsy has an NPV, and PPV of 100%. Hysteroscopy with curettage had a sensitivity of 92.3% (95% CI: 77.8% - 100%), NPV of 96.4% (95% CI: 89.5% - 100%), and specificity and PPV of 100%.

**Fig 1:**
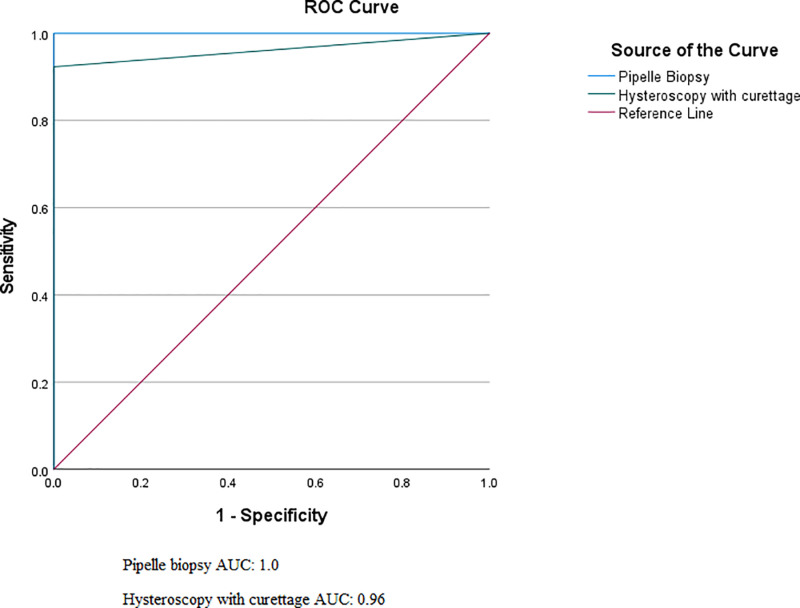
ROC Curve for pipelle biopsy compared to hysteroscopy with curettage.

McNemar’s test showed no significant difference between hysteroscopy with curettage and pipelle for CE detection (P = 1.0). Also, Stuart-Maxwell’s test was used to compare the diagnostic accuracy of pipelle and hysteroscopy with curettage, and there was no significant difference between them in their sampling accuracy (P = 0.317). According to ROC analysis, hysteroscopy had an AUC of 96.2% (95% CI: 87.6% - 100%), and pipelle had an AUC of 100% (Fig 1). Based on the results of our study, it can be said that pipelle biopsy and hysteroscopy with curettage both have excellent accuracy (AUC >  0.9), and none of them are superior to each other.

## Discussion

Based on the results of our study, the prevalence of CE in RIF patients can be estimated at 32.5% (±7.4%). Hysteroscopy with curettage and pipelle biopsy both have high diagnostic performance for the detection of chronic endometritis, with their sensitivity and specificity being 100% and 100% and 92.3% and 100, respectively; none of them were superior to each other in terms of diagnostic performance.

Several factors play a role in the process of implantation, which can be categorized into factors related to the embryo, those related to the endometrium and the general well-being of the mother, and those related to the technique used, in case ARTs are used. Embryonic causes for implantation failure include parental chromosomal abnormalities, inadequate sperm or oocyte quality, and those of embryonic quality. Karyotyping can be of use in detecting the cause of infertility when parental chromosomal abnormalities are present. Sperm and oocyte quality are also controlled in the process of ARTs; embryonic quality can also be controlled using preimplantation genetic screening, commonly known as PGS or PGT.

Etiologies related to maternal well-being vary tremendously; therefore, they are mostly categorized based on the organ system involved. Hormonal imbalances are one of the subgroups of maternal reproductive burdens affecting fertility; patients with polycystic ovary syndrome (PCOS) are known to have higher rates of infertility and implantation failures [7,15].

Coagulation disorders such as antiphospholipid syndrome (APS) and factor V Leiden can also deteriorate implantation rates. Certain habits in a patient’s lifestyle can also contribute to RIF; obesity (by insulin resistance), stressful life, and excessive smoking have been known to decrease implantation rates [15,16,35–37].

Uterine abnormalities are another group of disorders jeopardizing implantation, including uterine polyps, fibroids, adenomyosis, hydrosalpinx, and endometriosis [7,15]. Immunological dysregulations in the endometrium can contribute to implantation failure in RIF patients; increased uterine natural killer (uNK) cells, decreased Treg cells, and Th1 > Th2 cell count have also been noted in studies as immunological indicators in RIF patients [4,5,7,8,17].

Endometrial disorders can severely impact endometrial receptivity, which can be simply defined as the capability of the endometrium to attach, nurture, and culminate the embryo. Infection or inflammation in the endometrial lining can narrow the fertility window or the few days in each menstruation cycle when the endometrium has the best receptivity for the embryo(s). Many studies have found that the prevalence of both acute and chronic endometritis is significantly higher in RIF patients [3,6–8,15,16,19,30,36,38]. Uterine sample cultures in RIF patients have shown that uterine infections and microbiome disturbances are significantly more common in RIF patients compared to healthy individuals; E. coli, Streptococci, Staphylococci, E. faecalis, and yeast species are the most commonly found pathogens [3,6,7,15]. A meta-analysis study by Vitagliano et al. has shown that when treated, live birth rates (LBR), implantation rates (IR), and clinically confirmed pregnancy rates (CPR), ongoing pregnancy rate (OPR) can significantly increase in RIF patients, but a confirmation biopsy is required for that. Without a biopsy to confirm CE treatment with antibiotics, no significant increase in IR, CPR, OPR, or LBR was observed, but when the treatment was confirmed with a follow-up biopsy, significant improvements in OPR/LBR (OR: 6.81, 95% CI: 2.08 - 22.24), CPR (OR: 4.02, 95% CI: 1.35 – 11.94), and IR (OR: 3.24, 95% CI: 1.33 – 7.88) were achieved. There was no significant difference between non-CE patients and patients whose treatment was confirmed in OPR/LBR (OR: 0.7, 95% CI: 0.08 – 5.86), CPR (OR: 1.09, 95% CI: 0.29 – 4.14), and IR (OR: 1.05, 95% CI: 0.32 – 3.47) [19].

Endometrial thinning also jeopardizes implantation; studies have estimated that an endometrial thickness (ET) of at least 6-8 mm in the proliferative phase is required for successful implantation [39]. Different treatments have been used for this matter. Estrogen and progesterone support are used mostly to increase ET. Some studies have evaluated the use of platelet-rich plasma (PRP) and granulocyte colony-stimulating factor (G-CSF) to increase ET; PRP infusions increased ET by 1.0 mm (95% CI: 0.5 – 1.7) compared to previous cycles, CPR (OR: 1.43, 95% CI: 1.15 – 1.86) and LBR (1.27, 95% CI: 1.14 – 1.51) were significantly enhanced by PRP infusion in RIF patients [40]. A meta-analysis conducted by Fu et al. showed that G-CSF administration increased IR (RR =  2.51; 95% CI: 1.82, 3.47) and CPR (RR =  1.93; 95% CI: 1.63, 2.29) in RIF patients [41].

Treating CE can enhance implantation rates, but there is still controversy on whether treating CE can increase the implantation rates in RIF patients to that of the normal population [3,42]. Diagnosing CE, on the other hand, is a difficult matter; CE is often clinically silent, and up to now, there is still no consensus in studies investigating CE on the exact definition and diagnostic criteria. A meta-analysis by Huang et al. has shown that different studies use a number of one to five plasma cells per HPF to diagnose CE [33]. This massive heterogeneity in studies can have a disastrous effect on future studies and clinical practice since it can lead to false positives and negatives and bias future study results. That aside, plasma cell count in endometrial samples can be biased since many different factors can mistake the pathologist; mononuclear cell infiltrates, plasmacytoid stromal cells, abundant mitoses in the endometrial stroma, pre-decidual reactions in the late secretory phase, exogenous progesterone treatment changes, and even some menstrual changes can all mislead the pathologist to report a higher number of plasma cells [3,6,33].

Recent studies have used immunohistochemical methods to increase the accuracy of plasma cell counts further; CD138 (Syndecan-1) staining has been used in recent studies; CD-138 staining in 107 RPL patients found a significantly higher prevalence of CE compared to morphology and HE staining alone (56% vs. 13%, P < 0.01) [43]. Timing of sampling can also lead to different results; Liu et al. collected uterine samples exactly seven days after the LH surge, but most studies collected samples in the proliferative phase, which can be a confounding factor when interpreting results [8]. The role of hysteroscopy has been assessed in a study by Bouet et al.; they estimated the sensitivity of hysteroscopy compared to CD-138 staining to be 40% and concluded that an office hysteroscopy is a useful tool that can be used in adjunction with histopathological methods [3]. Hysteroscopy with biopsy is another method that has been investigated in studies; Vitale et al. have compared snake, alligator, and spoon hysteroscopic forceps in their sampling quality [44]. No differences were observed between them in terms of duration of biopsy (P =  0.334), number of attempts (P = 0.602), and biopsy appropriateness (P =  0.592). Spoon forceps use led to higher levels of pain (P <  0.001) compared to the other two and received lower scores by operators [44]. Blind methods of sampling have also been compared in a study by Abdelazim et al. Pipelle biopsy had 100% sensitivity, specificity, and accuracy in diagnosing endometrial hyperplasia, carcinoma, proliferative and secretory endometrium. Pipelle biopsy had a sensitivity of 88.9%, 60% and accuracy of 99.3% and 98.6% for diagnosing endometritis and polyps, respectively [45]. Hysteroscopy is however preferred in some scenarios; it enables the surgeon to visualize the uterine cavity, making it easier to detect the structural abnormalities in some cases, mostly complicated ones. It also provides the surgeon with the ability to have immediate interventions, which the pipelle biopsy does not. In some cases the pipelle biopsy might also provide us with inadequate samples, in which hysteroscopy might help [12].

Our study is the first one to compare these two methods and show that pipelle and hysteroscopy with curettage have a similar diagnostic performance in diagnosing CE. Pipelle biopsy is a cost-effective outpatient method that does not require the patient to undergo anesthesia and does not have the risks of perforation, bleeding, and infection as opposed to curettage, which is currently the best sampling method for CE diagnosis. Curettage can be even considered to be harmful in patients suffering from primary infertility.

## Limitations

Our study has some limitations. Our suggestion for future studies is to use IHC staining methods and uterine sample cultures to further increase their accuracy and measure the post-treatment RIF prevalence using a confirmation biopsy. Due to our stringent inclusion criteria and limited patient recruitment time, we had a small sample size that could compromise the generalizability of our results. Our study design, in which the patients underwent pipelle biopsy and two days later, underwent biopsy using curettage, might have introduced an interval bias for the diagnostic accuracy of curettage, undermining its diagnostic accuracy since there was a short period of time between the samplings. However, since there is considerable variation in the endometrium each month, delaying the sampling for a month was not reasonable.

## Conclusion

Although CE treatment does not increase the implantation rate in RIF patients to that of the normal population, it enhances them significantly. If correctly diagnosed and treated, CE treatment can help RIF patients by increasing their endometrial receptivity, but it is crucial that treatment is confirmed using a follow-up biopsy.

## Supporting information

S1 Data Pipelle data. (XLSX)

## Abbreviations

ANOVA: Analysis of Variance
APS: Antiphospholipid Syndrome
ART: Assistive Reproductive Techniques
AUC: Area Under the Curve
BMI: Body Mass Index
CPR: Clinically Confirmed Pregnancy Rates
CPP: Chronic Pelvic Pain
D&C: Dilation and Curettage
ET: Endometrial Thickness
G-CSF: Granulocyte Colony-Stimulating Factor
GIFT: Gamete Intrafallopian Transfer
HPF: High-Power Field
ICSI: Intracytoplasmic Sperm Injection
IR: Implantation Rates
IVF: In Vitro Fertilization
K-S Test: Kolmogorov-Smirnov Test
LBR: Live Birth Rates
OPR: Ongoing Pregnancy Rate
PCOS: Polycystic Ovary Syndrome
PGT: Preimplantation Genetic Testing
PRP: Platelet-Rich Plasma
ROC: Receiver Operating Characteristic
RPL: Recurrent Pregnancy Loss
SD: Standard Deviation
Th Cell: T Helper Cell
Treg Cell: Regulatory T Cell
uNK: Uterine Natural Killer
ZIFT: Zygote Intrafallopian Transfer.
